# 432. Predictors for oxygen requirements in patients hospitalized with COVID-19 during the Omicron surge, January 2022

**DOI:** 10.1093/ofid/ofad500.502

**Published:** 2023-11-27

**Authors:** Jonathan K Pinsky, Mary Anderson, Ines M Casey, Jason M Pinsky

**Affiliations:** Edward Hospital, Naperville, IL, Park Ridge, Illinois; Edward Hospital, Naperville, IL, Park Ridge, Illinois; Metro Infectious Disease Consultants, Naperville, IL., Naperville, Illinois; Midwestern University, Downers Grove, IL, Chicago, Illinois

## Abstract

**Background:**

Omicron replaced Delta as the predominant SARS-CoV-2 variant during December 2021. The period of escalating hospitalizations in January 2022 is a window to study the impact of vaccination on disease severity.

Edward Hospital COVID admissions & CDC Nowcast variant proportions during January 2022

**Figure:**
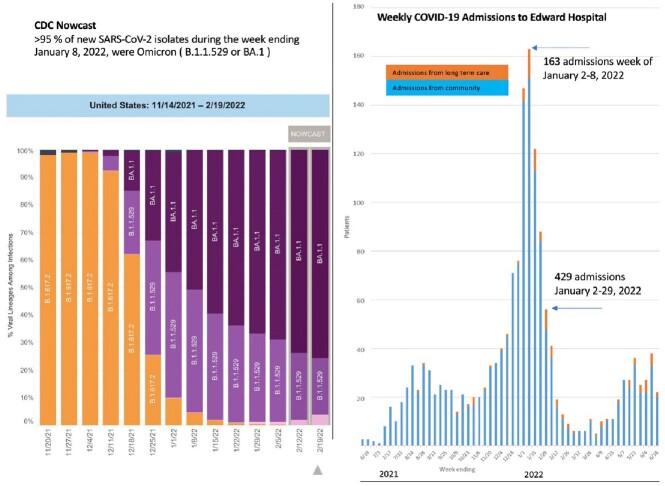

Left: CDC Nowcast showing Omicron accounted for >95% new infections week ending January 8, 2022 , Right: Edward Hospital COVID admissions from 6/2021-6/2022. Edward Hospital is a 359 bed community hospital in Naperville, IL.

**Methods:**

Retrospective chart review of adults admitted to Edward Hospital medical units or ICU with COVID during January 2022, for risk factors: age, body mass index (BMI), immunosuppression, gender, COVID vaccination, and medical conditions as predictors for disease severity, measured by level of supplemental oxygen requirements (O2) maintained > 24 hours. Outcomes were (1) any O2 if not on home O2, (2) high level O2 [ high flow (HF), noninvasive (NIV), or mechanical ventilation (MV)], and (3) hospital death or MV.

Methods

**Figure:**
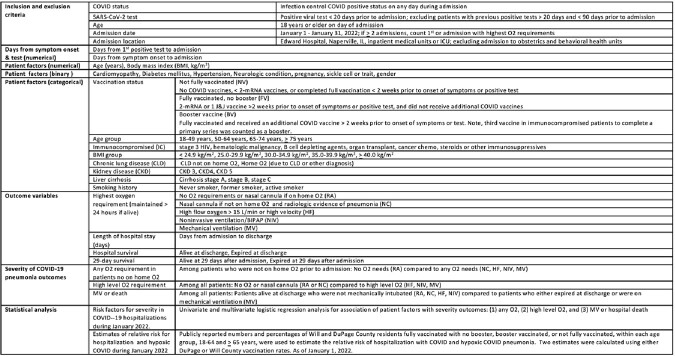

Input and output variables measured in retrospective chart review

**Results:**

O2 requirements were (1) any O2 in 133 of 379 admissions not on home O2 and (2) high level in 58 of 393 all admitted. 28 died, 15 on MV. Vaccination was within 5 months in 83 of 85 who had boosters and 16 of 144 who did not. In a multivariate analysis, factors associated with (1) any O2, (2) high level O2, and (3) hospital death or MV, (odds ratio [95%CI]), were being not fully vaccinated (7.86 [3.78-16.4], 5.17 [2.01-13.3], 5.21 [1.63-16.6]) compared to booster vaccinated, immunocompromised (2.79 [1.38-5.63]), 3.79 [1.66-8.67], 4.63 [1.72-12.5]), and age > 75 (2.85 [1.34-6.07]), 4.02 [1.47-11.2], 5.68 [1.49-21.7] ) compared to 50-64 years. Other factors associated with any O2 were BMI > 35 kg/m2 {35.0-39.9 (2.4 [1.09-5.26]), > 40.0 (3.29 [1.34-8.1]) compared to < 24.9} and age 65-74 (2.13 [1.03-4.38]) compared to 50- 64 years; with high level O2 were cardiomyopathy (2.11 [1.04-4.32) and male gender (1.93 [1.01-3.7]); with MV or death were cardiomyopathy (2.67 [1.11-6.42]) and CKD (3.17 [1.27-7.96]). Association was not found with (1), (2), or (3) for no booster if vaccinated (1.07 [0.522-2.21], 1.08 [0.415-2.81], 0.992 [0.328-3.0]), age 50-64 compared to 18-54 years (1.04 [0.495-2.2], 1.2 [0.418-3.43], 0.355 [0.037-3.75]), or other conditions.

Multivariate analysis for factors associated with any O2 requirements in patients not on home O2

**Figure:**
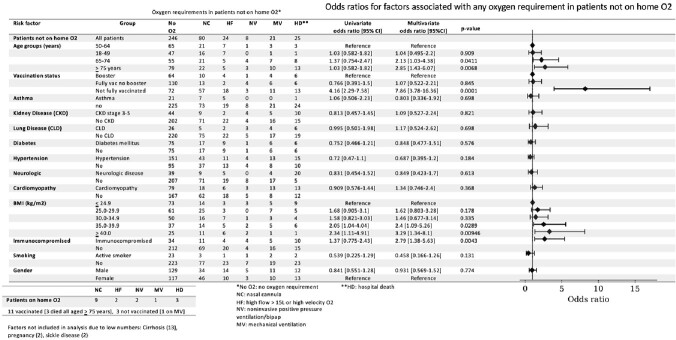

Top: Oxygen requirements and hospital deaths for patients not on hone O2, Multivariate analysis for risk factors associated with any O2 requirements in patients not on home O2 Bottom: O2 requirements and hospital deaths for patients on home O2

Multivariate analysis for factors associated with high level O2 and the composite of MV or hospital death

**Figure:**
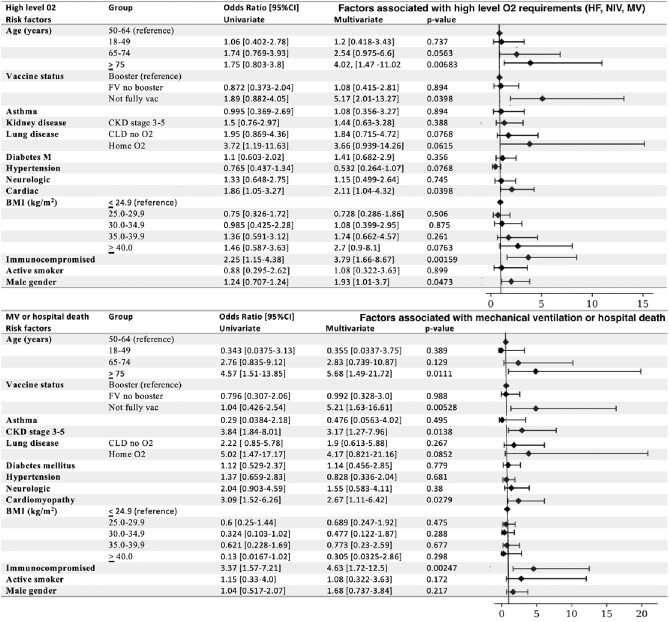

Top: Factors associated with high level O2 (high flow, noninvasive or mechanical ventilation) Bottom: Factors associated with mechanical ventilation or hospital death

Secondary outcomes and associations

**Figure:**
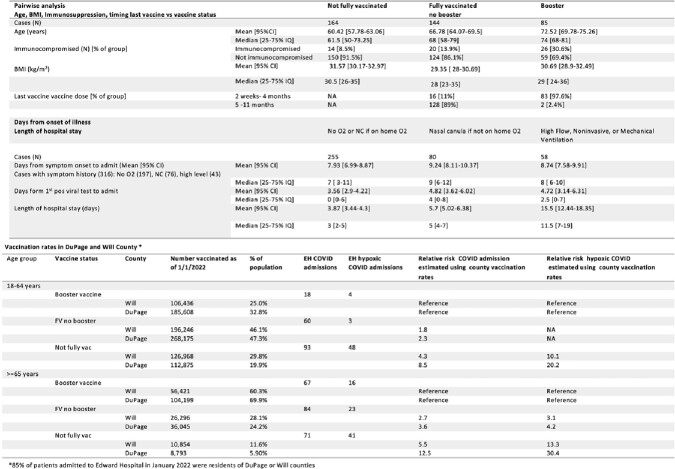

Top: Pairwise association of vaccination status with age, BMI, immunosuppression, and time since last vaccine Bottom: Vaccination rates by age groups in Will and DuPage counties and estimation of relative risk using percentage vaccinated for each age group

**Conclusion:**

Risk factors for hypoxia in patients hospitalized with COVID during the Omicron surge were not being fully vaccinated, immunosuppression, age > 65 years, and BMI > 35 mg/kg^2^. Boosters were not associated with less severe outcomes in vaccinated cases, suggesting immune memory plays a major role in averting severe disease.

Summary

**Figure:**
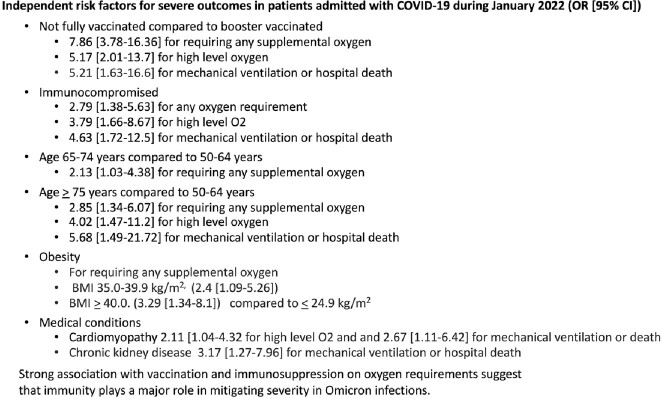

Conclusions

**Figure:**
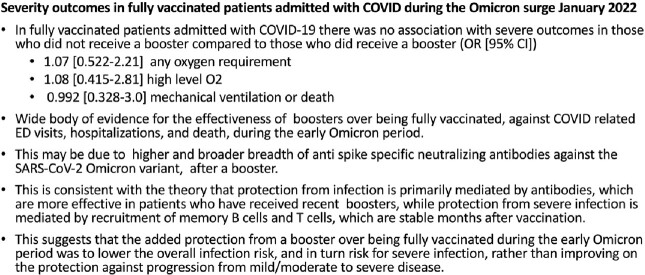

Literature

**Figure:**
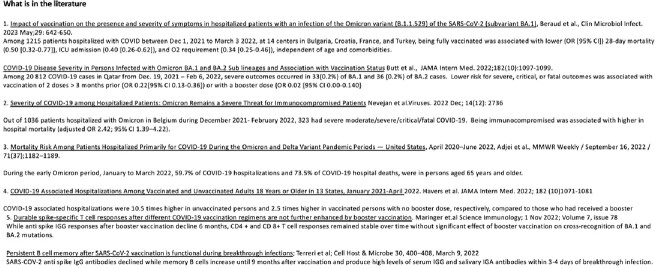

**Disclosures:**

**All Authors**: No reported disclosures

